# Supplementary material for: miR-10a-5p Regulates the Proliferation and Differentiation of Porcine Preadipocytes Targeting the KLF11 Gene
Source: Animals (Basel). 2024 Jan 22;14(2):337. doi: 10.3390/ani14020337 (PMC10812476; doi:10.3390/ani14020337)
Supplement: Supplementary file 1 [file animals-14-00337-s001.zip › animals-2793703-supplementary.pdf]

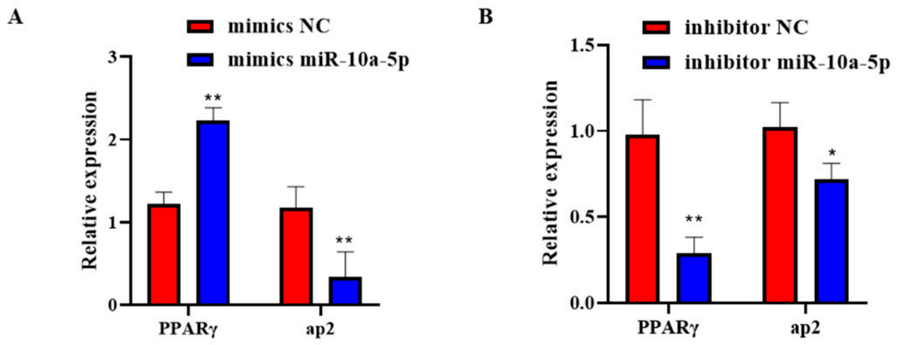

Figure S1: Western blotting gray value statistics. (A) Protein levels of PPAR γ and ap2 of adipogenic markers in preadipocytes following miR-10a-5p mimics. (B) Protein levels of PPAR γ and ap2 of adipogenic markers in preadipocytes following miR-10a-5p inhibitor.

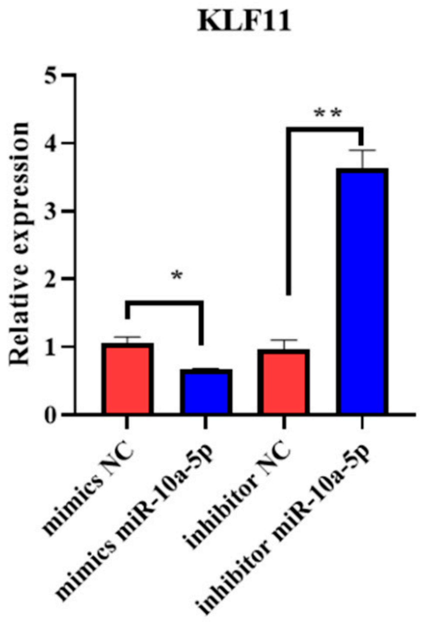

Figure S2: Western blotting gray value statistics of KLF11 protein expression in porcine preadipocytes following transfection with miR-10a-5p mimics or inhibitor.

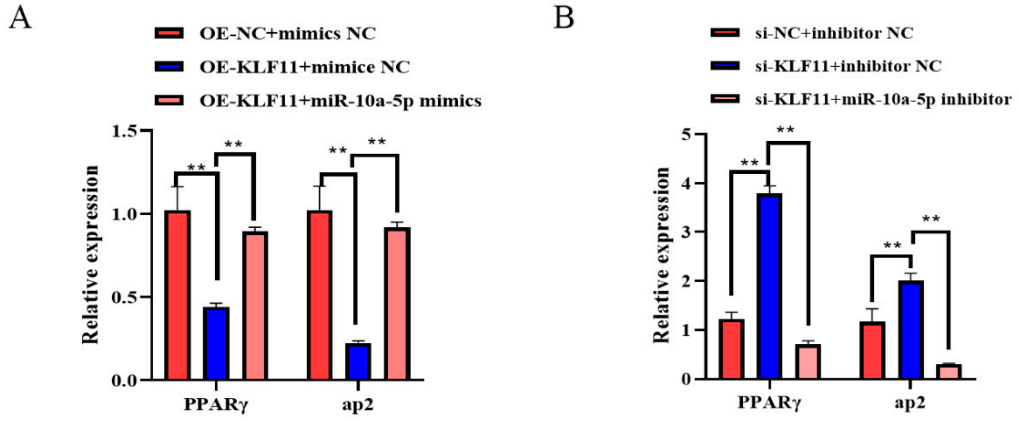

Figure S3: Western blotting gray value statistics. (A) Protein levels of PPAR γ and ap2 of adipogenic markers in preadipocytes following transfected with OE-KLF11 or miR-10a-5p mimic. (B) Protein levels of PPAR γ and ap2 of adipogenic markers in preadipocytes following transfected with si-

KLF11 or miR-10a-5p inhibitor.
